# Benzodiazepine prescribing in tunisia: A study about psychiatrists prescribing habits

**DOI:** 10.1192/j.eurpsy.2021.1292

**Published:** 2021-08-13

**Authors:** M. Lagha, U. Ouali, F. Nacef

**Affiliations:** Department Of Psychiatry A, Razi hospital, Manouba, Tunisia

**Keywords:** Benzodiazepines, Prescribing, psychiatry, habits

## Abstract

**Introduction:**

Benzodiazepines (BZDs) are psychotropic drugs that are predominantly prescribed in psychiatry and that can have serious side effects. BZDs prescribing is well codified by several guidelines, yet the epidemiological data on their prescribing remains alarming.

**Objectives:**

Our study aimed to evaluate the general modalities of BZDs prescribing in psychiatry in Tunisia.

**Methods:**

This is a descriptive cross-sectional study conducted through a Google-forms self-administered questionnaire, intended for psychiatrists and psychiatric residents, over a period of two months, from April 1 to May 31, 2019.

**Results:**

One hundred physicians practicing in psychiatry answered our questionnaire. The response rate was 28%. The purpose of treatment with BZDs was explained to the patient at its initiation in 98.2% of cases and the risks associated with it in 87.7% of cases. Special precautions were taken in elderly patients (96.5%), at risk of respiratory failure (94.7%), and in cases of personality disorders (80.7%). Only three quarters of physicians took precautions before prescribing BZDs to a pregnant woman (77.9%). In cases of rebellious or refractory symptoms, 14.4% of the participants stated that they combine two BZDs. Before reproducing/repeating a BZD prescription, 18.4% of the participants indicated that they did not systematically and regularly assess its necessity.

**Conclusions:**

The severity of the side effects associated with BZDs, especially those of tolerance and dependence, are at the origin of strict prescribing rules, dictated by several guidelines. According to the results of our study and to the literature data, the prescribing practices of these molecules remain nonetheless in many cases non-compliant with the recommendations.

